# Implementation of an Antibiotic Stewardship Program in Long-term Care Facilities Across the US

**DOI:** 10.1001/jamanetworkopen.2022.0181

**Published:** 2022-02-28

**Authors:** Morgan J. Katz, Pranita D. Tamma, Sara E. Cosgrove, Melissa A. Miller, Prashila Dullabh, Therese A. Rowe, Roy Ahn, Kathleen Speck, Yue Gao, Savyasachi Shah, Robin L. P. Jump

**Affiliations:** 1 Department of Medicine, Johns Hopkins University School of Medicine, Baltimore, Maryland; 2 Johns Hopkins University School of Medicine, Baltimore, Maryland; 3 Center for Quality Improvement and Patient Safety, Agency for Healthcare Research and Quality, Rockville, Maryland; 4 NORC at the University of Chicago, Bethesda, Maryland; 5 Northwestern Feinberg School of Medicine, Chicago, Illinois; 6 NORC at the University of Chicago, Chicago, Illinois; 7 Armstrong Institute for Patient Safety and Quality, Johns Hopkins University School of Medicine, Baltimore, Maryland; 8 Geriatric Research Education and Clinical Center, VA Northeast Ohio Healthcare System, Cleveland, Ohio; 9 Case Western Reserve University School of Medicine, Cleveland, Ohio

## Abstract

**Importance:**

Antibiotic overuse in long-term care (LTC) is common, prompting calls for antibiotic stewardship programs (ASPs) designed for specific use in these settings. The optimal approach to establish robust, sustainable ASPs in LTC facilities is unknown.

**Objectives:**

To determine if the Agency for Healthcare Research and Quality (AHRQ) Safety Program for Improving Antibiotic Use, an educational initiative to establish ASPs focusing on patient safety, is associated with reductions in antibiotic use in LTC settings.

**Design, Setting, and Participants:**

This quality improvement study including 439 LTC facilities in the US assessed antibiotic therapy data following a pragmatic quality-improvement program, which was implemented to assist facilities in establishing ASPs and with antibiotic decision-making. Training was conducted between December 2018 and November 2019. Data were analyzed from January 2019 to December 2019.

**Interventions:**

Fifteen webinars occurred over 12 months (December 2018 to November 2019), accompanied by additional tools, activities, posters, and pocket cards. All clinical staff were encouraged to participate.

**Main Outcomes and Measures:**

The primary outcome was antibiotic starts per 1000 resident-days. Secondary outcomes included days of antibiotic therapy (DOT) per 1000 resident-days, the number of urine cultures per 1000 resident-days, and *Clostridioides difficile* laboratory-identified events per 10 000 resident-days. All outcomes compared data from the baseline (January-February 2019) to the completion of the program (November-December 2019). Generalized linear mixed models with random intercepts at the site level assessed changes over time.

**Results:**

Of a total 523 eligible LTC facilities, 439 (83.9%) completed the safety program. The mean difference for antibiotic starts from baseline to study completion per 1000 resident-days was –0.41 (95% CI, –0.76 to –0.07; *P* = .02), with fluoroquinolones showing the greatest decrease at –0.21 starts per 1000 resident-days (95% CI, –0.35 to –0.08; *P* = .002). The mean difference for antibiotic DOT per 1000 resident-days was not significant (–3.05; 95% CI, –6.34 to 0.23; *P* = .07). Reductions in antibiotic starts and use were greater in facilities with greater program engagement (as measured by webinar attendance). While antibiotic starts and DOT in these facilities decreased by 1.12 per 1000 resident-days (95% CI, –1.75 to –0.49; *P* < .001) and 9.97 per 1000 resident-days (95% CI, –15.4 to –4.6; *P* < .001), respectively, no significant reductions occurred in low engagement facilities. Urine cultures per 1000 resident-days decreased by 0.38 (95% CI, –0.61 to –0.15; *P* = .001). There was no significant change in facility-onset *C difficile* laboratory-identified events.

**Conclusions and Relevance:**

Participation in the AHRQ safety program was associated with the development of ASPs that actively engaged clinical staff in the decision-making processes around antibiotic prescriptions in participating LTC facilities. The reduction in antibiotic DOT and starts, which was more pronounced in more engaged facilities, indicates that implementation of this multifaceted program may support successful ASPs in LTC settings.

## Key Points

**Question:**

Is framing antibiotic stewardship as a patient safety issue and emphasizing direct care staff engagement associated with reductions in antibiotic use across US long-term care facilities?

**Findings:**

In this quality improvement study including 439 long-term care facilities, participation in training on antibiotic stewardship from the Agency for Healthcare Research and Quality (AHRQ) was associated with a reduction in antibiotic use and urine culture collection. Fluoroquinolones, an antibiotic class targeted by the AHRQ safety program, had the greatest decrease.

**Meaning:**

These results suggest that the AHRQ safety program provides a pragmatic framework for the development of antibiotic stewardship programs in long-term care facilities that may reduce antibiotic use.

## Teaser

This quality improvement study examines the association between long-term care facility participation in an antibiotic reduction educational program and antibiotic use among facility residents.

## Introduction

Antibiotic use is pervasive in long-term care (LTC) settings. Over 70% of LTC residents may receive at least 1 course of antibiotics annually, with 40% to 75% of prescriptions deemed to be inappropriate or not concordant with guidelines.^1,2^ As a component of widespread efforts to reduce threats posed by antimicrobial-resistant organisms across the spectrum of health care settings, the Centers for Medicare & Medicaid Services (CMS) requires that LTC facilities establish antibiotic stewardship programs (ASPs).^3^ While there is sufficient experience and evidence to guide antibiotic stewardship implementation in hospital environments,^4^ similar efforts in LTC settings are still in the early stages of development.^5^

A 2017 review^6^ of antimicrobial stewardship interventions in LTC settings concluded that effective ASPs included those that incorporated multidisciplinary education with enduring material (ie, educational modules that can be used over time and in different locations) as well as tools that integrate antimicrobial stewardship principles into the workflow patterns usually present in LTC settings. Useful educational material and tools for nurses and nurse assistants can aid in the gathering of appropriate clinical data (eg, symptoms, vital signs) and the effective communication of findings with prescribers; for prescribers, these interventions can improve decision-making regarding indications to start and stop antibiotics after review of residents’ diagnostic test results and clinical status. These approaches informed the development and implementation of the Agency for Healthcare Research and Quality (AHRQ) Safety Program for Improving Antibiotic Use, an initiative to establish ASPs specific to LTC settings.

The overarching goals of the AHRQ Safety Program were to establish sustainable ASPs that improve the use of antibiotics and enhance patient safety. Specifically, in LTC settings the safety program provided frontline nursing staff and prescribers with tools to incorporate antibiotic stewardship principles into routine decision-making by (1) emphasizing the importance of recognizing signs and symptoms that herald common infectious syndromes in LTC residents and (2) underscoring the importance of communication around antibiotic prescribing with clinical staff as well as residents and caregivers. The foundation of the safety program was drawn largely from the Comprehensive Unit-based Safety Program methodology, a patient safety approach that emphasizes the importance of improved teamwork, clinical best practices, and the science of safety.^7^ In this quality improvement study, we examined the implementation of the AHRQ Safety Program in 439 LTC facilities across the US. We assessed process measures related to participation by staff and their association with antibiotic stewardship practices in LTC settings, such as rates of antibiotic use and total urine culture and *Clostridioides difficile* tests sent.

## Methods

The AHRQ Safety Program was a quality improvement initiative aimed to assist LTC facilities in establishing antibiotic stewardship programs by addressing both facility culture and technical knowledge around antibiotic prescribing. The program was conducted from December 2018 to November 2019; changes in antibiotic use and process measures were compared in participating facilities from the beginning to the end of the program using a pre-post design. The AHRQ Safety Program was exempted from review and informed consent requirements by the institutional review board at the Johns Hopkins University School of Medicine as research that did not involve human participants. All methods and analysis were in compliance with the Standards for Quality Improvement Reporting Excellence Standards for Quality Improvement Reporting Excellence (SQUIRE) version 2.0 reporting guideline.

### Enrollment Criteria

Eligible LTC sites included nursing homes and the following facility types: skilled nursing care, hospice, dementia care, and residential and continuing care communities. Stand-alone long-term acute care hospitals as well as assisted living, adult day care, rehabilitation, pediatric skilled nursing facilities, and developmentally disabled community settings were excluded.

The predetermined maximum enrollment was 500 sites; in anticipation of attrition, additional interested sites were permitted to be on a waitlist through the first 3 months of the safety program. Each site identified at least 1 champion, defined as an individual dedicated to facilitating change and supporting improvements, usually the assistant director or director of nursing. The sites also identified an antibiotic stewardship team, usually consisting of members of the quality assurance and performance improvement program. In addition to the antibiotic stewardship team, all health care workers were encouraged to be involved in the safety program and were given access to the project website, including nurses, nurse assistants, advanced practice practitioners, and physicians. Continuing education credits were provided for participants who attended live webinars.

### Safety Program Content

The LTC safety program adapted the Four Moments of Antibiotic Decision Making framework, previously developed for acute care settings,^8,9^ to the LTC setting. The Four Moments framework encourages frontline staff to address 4 critical questions around antibiotic decision-making (Box). This framework was emphasized over the 12-month intervention period, which included 15 webinars along with an array of content and tools such as narrated presentations, posters, and pocket cards (eTable 1 in the Supplement). Webinars were repeated 3 times at different times of day and recorded for participants. The first 7 webinars introduced ASP development, data collection, and the science of safety as it applies to antibiotic use among LTC residents. Five webinars addressed technical aspects of caring for residents with possible infections. The final 3 webinars focused on communication and sustainability. LTC infectious disease experts led all webinars, each of which was recorded for later viewing; slides and a script were also available on the safety program website.

Box. **The 4 Moments of Antibiotic Decision Making**

**1. Make the Diagnosis**

Does the resident have symptoms that suggest an infection?

**2. Cultures and Empiric Therapy**

What type of infection is it? Have we collected appropriate cultures before starting antibiotics? What empiric therapy should be initiated?

**3. Duration of Therapy**

What duration of antibiotic therapy is needed for the resident’s diagnosis?

**4. Stop, Narrow, Change to Oral**

It’s been 2-3 days since we started antibiotics. Reevaluate the resident and review results of diagnostic tests. Can we stop antibiotics? Can we narrow therapy? Can we change to oral antibiotics?

### Support Available to Participating Sites

Participants had access to the safety program project team throughout the year via question-and-answer sessions following webinars, twice-monthly office hours led by LTC infectious disease experts, and an email account for additional questions. An external quality improvement expert well-versed in large-scale implementation programs^10^ was assigned to each site. These experts offered additional assistance with implementing the safety program and collecting data, interacting by telephone with each site monthly or whenever a facility had questions, reduced engagement, or issues with uploading data or project implementation.

### Data Collection

The primary outcome of the safety program we examined was antibiotic starts per 1000 resident-days. Monthly antibiotic use data were submitted for 16 intravenous (IV) and 25 oral antibiotics from January 2019 to December 2019, beginning the month following initiation of the safety program. Secondary outcomes included days of antibiotic therapy (DOT) per 1000 resident-days, total urine cultures collected per 1000 resident-days, and the number of facility-onset *C difficile* laboratory-identified (LabID) events per 10 000 resident-days (eMethods 1 in the Supplement). Individualized quarterly data reports were provided to each site to assess progress over the course of the program and to compare facilities (eMethods 2 in the Supplement). A survey assessed the characteristics of the participating sites and gathered information about domains relevant to antibiotic stewardship at baseline and program completion.^11^ Process measures for the intervention included the number of unique downloads of materials from the program website, attendance, continuing education credits claimed, and participants ratings of the webinars.

### Data Integrity

Several steps were followed to ensure valid data collection. An informational onboarding webinar at the start of the safety program provided detailed instructions regarding data collection, and a standardized template was used to collect and upload data (eMethods 1 in the Supplement). Sites unfamiliar with electronic data extraction were paired with sites that were successful in navigating the same electronic health record system to access antibiotic use data. Sites without electronic health record systems were advised on accurate manual collection of data. The quality improvement expert assigned to each facility provided regular check-ins and assisted with data collection issues. The monthly data provided by each participating site was assessed and compared with its previous submissions to assess for internal consistency. Sites reporting antibiotic use rates, total urine cultures, or *C difficile* LabID events significantly higher or lower than expected when compared with either their individual historical data or the range of values obtained for the cohort were identified and contacted to review and reextract data when needed.

### Statistical Analysis

We used a pre-post design and compared the primary and secondary outcomes between the baseline (January 2019) and program completion periods (December 2019). Outcome measures were compared from the first 2 baseline months with the outcome measures from each subsequent bimonthly period to illustrate how the association of program training with outcomes evolved over the implementation period; engagement of facilities was measured by level of attendance of webinars (ie, at least 1 person in facility attending each session) or educational credits claimed. Facilities were classified as high for engagement if more than 8 of 15 webinars were attended or an educational credit was claimed, low if 1 to 7 webinars were attended (or credit was claimed), and no engagement if zero webinars were attended (or credit claimed).

The unit of analysis was the site, and the random effect analyzed was site-level. We used generalized linear mixed models assuming negative binomial distribution with a random intercept for LTC facility because there were zero prescriptions for certain antibiotic classes across months. Data analyses were conducted using SAS version 9.4 (SAS Institute Inc) and Stata version 16.1 (StataCorp LLC). Findings were considered significant at *P* < .05 with 2-sided testing.

## Results

### Enrollment

A total of 439 of 523 sites (83.9%) remained in the AHRQ Safety Program at the end of the 1-year period; mean (SD) bed capacity of these sites was 124.3 (96.0) beds. Common reasons for withdrawal included time limitations (22 sites), staff turnover (21 sites), or both (6 sites) (eFigure 1 in the Supplement). A total of 246 facilities (56.0%) included were part of a larger health care system, 133 facilities (30.3%) were not owned by a larger health care system, and 60 (13.7%) were hospital-based facilities (Table 1). eFigure 2 in the Supplement illustrates the distribution of retained LTC sites across the US.

**Table 1.** Characteristics of Participating Long-term Care Sites

Characteristics	Sites, No. (%) (N = 439)
Certified beds in facility, mean (SD) [range]	124.3 (96.0) [18-874]
Bed capacity	
0-74	106 (24.1)
75-149	229 (52.2)
≥150	104 (23.7)
Ownership	
Hospital-based	60 (13.7)
Nonhospital–based	
Owned by a larger system	246 (56.0)
Not owned by a larger system	133 (30.3)
Proportion of residents in short-stay beds	
<25%	233 (53.1)
≥25% and <50%	70 (15.9)
≥50% and less than 75%	45 (10.3)
≥75%	91 (20.7)
Community setting	
Urban	108 (24.6)
Suburban	156 (35.5)
Rural	175 (39.9)

### Participation

The AHRQ Safety Program website had 1879 unique users with over 4000 unique downloads of posted materials, averaging 200 downloads per item (eFigure 3 in the Supplement). There was a mean of 230 attendees (range, 86-440) for each of the 15 webinar topics and a mean of 51 participants (range, 21-118) for each of the 24 office hour sessions. Each webinar provided 0.5 medical education credits for physicians and 0.5 contact hours for nurses; participants claimed a total of 1361 credits. On average, 80% of participants described the webinars as “very useful” and 20% as “somewhat useful” (eFigure 4 in the Supplement). The safety program help desk received 2273 initial email inquiries from participants.

### Infrastructure

At the beginning of the safety program, infection preventionists at 363 (82.7%) and medical directors of 273 of 439 sites (62.2%) described themselves as being involved in antibiotic stewardship activities at their LTC facilities. By the end of the program, participation levels increased to 341 (92.9%; *P* < .001) and 257 of 367 participating sites (70.0%; *P* = .02), respectively. Actions to improve antibiotic use and antibiotic use tracking significantly increased throughout the course of the safety program. Required in-service training for nurses on topics related to antibiotic use increased from 328 of 439 sites (74.7%) at the start of the safety program to 331 of 367 sites (90.2%) at the end (*P* < .001). Comparing the beginning and the end of the safety program, sites were more likely to develop antibiotic prescribing recommendations (213 of 439 sites [48.5%] vs 205 of 367 [55.9%]; *P* = .04), perform postprescriptive review with feedback (166 [37.8%] vs 223 [60.8%]; *P* < .001), implement formulary restriction of antibiotics (66 [15.0%] vs 77 [21.0%]; *P* = .03), and track overall antibiotic use (384 [87.5%] vs 358 [97.5%]; *P* < .001) (Table 2).

**Table 2.** Characteristics of Antibiotic Stewardship Programs (ASPs) at Participating Long-term Care Sites at Baseline and End of Safety Program^a^

Assessed domains	Assessed items	Sites, No. (%)		*P* value
		Baseline (n = 439)	End of program (n = 367)	
Accountability	Infection prevention and control nurse involved with ASP	363 (82.7)	341 (92.9)	<.001
	Medical director involved with the ASP	273 (62.2)	257 (70.0)	.02
	Consultant pharmacist working at the facility	420 (95.7)	353 (96.2)	.71
Actions to improve antibiotic use	In-service training to nurses on topics related to antibiotic use	328 (74.7)	331 (90.2)	<.001
	Protocols for diagnosis and treatment of common infection syndromes	287 (65.4)	279 (76.0)	.001
	Antibiotic prescribing recommendations for facility	213 (48.5)	205 (55.9)	.04
	Working with the contracted laboratory to develop antibiogram	194 (44.2)	191 (52.0)	.03
	Postprescription review with feedback of select antibiotics	166 (37.8)	223 (60.8)	<.001
	Formulary restriction of some antibiotics	66 (15.0)	77 (21.0)	.03
	At least one of the above	402 (91.6)	362 (98.6)	<.001
	All activities above	28 (6.4)	41 (11.2)	.02
Antibiotic use tracking	Antibiotic starts	296 (67.4)	327 (89.1)	<.001
	Antibiotic DOT per 1000 resident-days	176 (40.1)	269 (73.3)	<.001
	Defined daily doses per 1000 resident-days	40 (9.1)	89 (24.3)	<.001
	At least one of the above tracking methods	384 (87.5)	358 (97.5)	<.001

Abbreviation: DOT, days of antibiotic therapy.

^a^ Compared with baseline, 367 of 439 (83.6%) of participating facilities completed the survey. Facilities that were neither hospital-based nor part of a larger system were more likely to complete the final assessment (126/133 facilities [94.7%] vs 51/60 [85%] for hospital-based facilities and 190/246 [77.2%] for facilities that are not hospital-based but part of a larger health system; *P* < .001), as were facilities with fewer than 25% short stays (205/233 facilities [88.0%] vs 162/206 [78.6%] for facilities with ≥25% short stay; *P* = .009).

### Antibiotic Use

A total of 410 of 439 participating facilities (93.4%) submitted comprehensive data for analysis of antibiotic use from the beginning to the end of the safety program. From baseline to the end of the program, antibiotic starts decreased from 7.9 to 7.5 per 1000 resident-days (–0.41 starts; 95% CI, –0.76 to –0.07; *P* = .02). Similarly, antibiotic DOT decreased from 64.1 to 61.0 per 1000 resident-days, although the difference was not significant (–3.1 DOTs; 95% CI, –6.3 to 0.23; *P* = .07) (Table 3). In a subanalysis based on webinar attendance, the 103 facilities attending at least 8 webinar sessions (ie, high-engagement facilities) saw the greatest reduction in antibiotic use. Antibiotic starts were reduced by 1.12 per 1000 resident-days (95% CI, –1.75 to –0.49; *P* < .001), from 8.3 to 7.2; differences in low (–0.29; 95% CI, –0.74 to 0.17) and no engagement facilities (0.40; 95% CI, –0.55 to 1.35) were not significant. DOT in high-engagement facilities was reduced by 9.97 per 1000 resident-days (95% CI, –15.37 to –4.56; *P* < .001), from 71.9 to 61.9; differences in low (–1.85; 95% CI, –6.07 to 2.37) and no engagement facilities (3.51; 95% CI, –6.73 to 13.75) were not significant (Table 4). The decrease in antibiotic starts and DOT use was most pronounced for LTC sites with more than 75% short-stay residents (eTables 2 and 3 in the Supplement). There was a significant reduction in antibiotic DOT for oral antibiotics (–2.80; 95% CI, –5.56 to –0.05, *P* = .046) but not in IV antibiotics (–0.03; 95% CI, –1.74 to 1.69, *P* = .98) across the entire cohort. Fluoroquinolone use decreased from 1.5 to 1.3 starts per 1000 resident-days (*P* = .002) and from 10.6 to 9.4 DOT per 1000 resident-days (*P* = .01) across all sites from the beginning to the end of the safety program (eTables 4 and 5 in the Supplement).

**Table 3.** Changes in Antibiotic Use, Urine Cultures Collected, and *Clostridioides difficile* LabID Events

Outcomes	Rate per 1000 resident-days		Difference (95% CI)	*P* value
	Baseline (n = 410)	End of program (n = 410)		
Antibiotic starts				
All antibiotics	7.89	7.48	–0.41 (–0.76 to –0.07)	.02^a^
Fluoroquinolones	1.49	1.28	–0.21 (–0.35 to –0.08)	.002^a^
Piperacillin-tazobactam	0.09	0.11	0.02 (–0.01 to 0.04)	.13
Third-generation cephalosporins	0.80	0.74	–0.06 (–0.14 to 0.02)	.15
Ceftazidime/cefepime	0.09	0.13	0.04 (–0.004 to 0.08)	.08
Antibiotic days of therapy				
All antibiotics	64.10	61.05	–3.05 (–6.34 to 0.23)	.07
Fluoroquinolones	10.6	9.41	–1.20 (–2.15 to –0.24)	.01^a^
Piperacillin-tazobactam	2.18	3.01	0.83 (–0.17 to 1.84)	.10
Third-generation cephalosporins	5.48	4.72	–0.76 (–1.44 to –0.88)	.03^a^
Ceftazidime/cefepime	1.41	2.19	0.78 (0.07 to 1.49)	.03^a^
Urine cultures collected	3.01	2.63	–0.38 (–0.61 to –0.15)	.001^a^
*Clostridioides difficile* LabID events/10 000 resident-days	1.66	1.50	–0.16 (–0.64 to 0.33)	.52

Abbreviation: LabID, laboratory-identified.

^a^ Indicates significant difference (*P* < .05) between the baseline (January-February 2019) and study completion periods (November-December 2019).

**Table 4.** Differences in Antibiotic Use Stratified by Facility Engagement

Engagement measure	Nursing homes, No. (%) (n = 439)	Difference per 1000 resident-days (95% CI)	
		Antibiotic starts	Antibiotic days of therapy
Webinars attended^a^			
None	82 (18.7)	0.40 (–0.55 to 1.35)	3.51 (–6.73 to 13.75)
Low (1-7)	254 (57.9)	–0.29 (–0.74 to 0.17)	–1.85 (–6.07 to 2.37)
High (≥8)	103 (23.5)	–1.12 (–1.75 to –0.49)^b^	–9.97 (–15.37 to –4.56)^b^
Webinars attended with educational credit claimed^a^			
None	295 (67.2)	–0.35 (–0.78 to 0.08)	–1.99 (–6.18 to 2.21)
Low (1-7)	83 (18.9)	–0.31 (–1.12 to 0.49)	–2.5 (–9.79 to 4.8)
High (≥8)	61 (13.9)	–0.77 (–1.62 to 0.07)	–7.92 (–14.93 to –0.92)^b^

^a^ Engagement measured by site based on a minimum of 1 participant.

^b^ Indicates a significant difference (*P* < .05) between the baseline (January-February 2019) and end of program periods (November-December 2019).

### Urine Cultures and *C difficile* LabID Events

The number of urine cultures per 1000 resident-days decreased from 3.0 to 2.6 (–0.38; 95% CI, –0.61 to –0.15; *P* = .001) between January 2019 and December 2019. There was no statistically significant change in the number of facility-onset *C difficile* LabID events per 10 000 resident-days across the safety program (–0.16; 95% CI, –0.64 to 0.32; *P* = .52). The reduction was significant between January and a study midpoint (May-June) (–0.57; 95% CI, –0.93 to –0.20; *P* = .002) (eTables 6 and 7, eFigure 5 in the Supplement).

## Discussion

The AHRQ Safety Program for Improving Antibiotic Use is the largest LTC antibiotic stewardship quality improvement initiative in the US to date. The safety program was associated with decreases in overall antibiotic starts, fluoroquinolone use, and urine cultures across a diverse cohort of LTC facilities across the country.

ASPs have been historically challenging to implement and sustain in LTC settings. Annual staff turnover is as high as 75% for direct care staff,^12^ and prescribing physicians are typically only onsite 1 to 2 times per week. LTC residents often have vague clinical symptoms or are nonverbal, making determining whether an infection is present challenging. Further, implementing data-driven quality improvement initiatives has particular hurdles; retention rates for implementation research in LTC are around 50%, largely because of high staff turnover rates and a lack of resources.^13,14^

The AHRQ Safety Program was designed to address unique challenges in the LTC setting and involve direct care staff in the antibiotic decision-making process. Most ASPs focus on educating physician prescribers in best practices or antibiotic prescribing guidelines. In LTC settings, nurses and nurse assistants are the backbone of the care system, serving as the point of contact between prescribers, family members, residents, and administration. Nurses and nurse assistants are well-positioned to serve as clear advocates for residents, particularly when it comes to patient safety and antibiotic prescribing practices, after receiving appropriate training in best practices around antibiotic prescribing.^15^ Antibiotic stewardship initiatives in LTC that have been multidisciplinary, involving both nurses and prescribers, have been associated with improvements in antibiotic prescribing practices in LTC settings.^16^ However, such programs have not directly involved nurse assistants, who perform the majority of direct resident care in the nursing home setting. The safety program created materials specifically targeted for all roles in the antibiotic decision-making process, including nurse assistants (eg, regarding sending appropriate microbiological cultures), nurses (on communicating infectious concerns with prescribers), family members (on talking with family members about antibiotic side effects), and program administration (on engaging senior executives). Engaging all staffing roles facilitates organizational culture change.

Reductions in antibiotic use were more pronounced in facilities with greater engagement in the program, suggesting that variation in uptake of this quality improvement initiative was associated with key outcomes. Furthermore, antibiotic use decreased significantly for oral antibiotics compared with IV antibiotics. Oral antibiotics are used much more frequently in LTC settings, and case scenarios presented on the webinars were largely directed toward reducing oral antibiotics. It is possible that the lack of change in IV antibiotic use may reflect courses of antibiotics started in the acute-care setting and continued in postacute care. Reducing fluoroquinolone use was a specific target of the safety program given the concerning adverse event profile of fluoroquinolones, particularly in older adults. Because of a combination of their oral bioavailability, broad spectrum, and ease of dosing, fluoroquinolones are frequently prescribed in LTC as first-line agents, even when alternatives with a more favorable side effect profile exist.^17^ There is a growing body of evidence in older adults describing adverse events with fluoroquinolone use including prolonged corrected QT intervals, tendinitis, aortic dissections, seizures, peripheral neuropathy, psychological disturbances, and *C difficile* infections (CDI).^18,19,20^ The AHRQ Safety Program educated prescribers and direct care staff on the potential side effects associated with fluoroquinolone use through case-based examples combined with guidance for safer alternative therapies for common infections encountered in LTC settings. We observed significant reductions in both antibiotic starts and DOTs for fluoroquinolones. Despite the association between fluoroquinolone use and CDI rates, we did not observe a reduction in CDI across sites.

Unnecessary treatment of asymptomatic bacteriuria is one of the primary reasons antibiotics are prescribed inappropriately in nursing homes.^21^ The common misperception by direct care staff or family members that changes in the appearance of urine (eg, malodorous, cloudy) indicate a urinary tract infection (UTI) can lead to obtaining unnecessary urine cultures to assess for UTI.^22^ Up to 50% of residents in nursing homes will have bacteria in their urine, and prescribers often feel inclined to treat a positive culture even if antibiotics are not indicated.^23^ We targeted misconceptions about when to test for and treat UTIs by educating direct caregivers about sending urine cultures only when specific signs and symptoms were present.^21^ We saw a significant reduction in the number of urine cultures ordered from the beginning to the end of the safety program, emphasizing that diagnostic stewardship has an important role in reducing antibiotic use in LTC facilities.

### Limitations

There are several limitations to this study. Because the AHRQ Safety Program was a quality improvement initiative and not a research study, we limited data collection to information necessary to improve antibiotic prescribing practices. Evaluation to determine if specific aspects of the safety program had a greater association with the primary outcomes were not assessed. Previous evidence supports implementation of the safety program as a bundle of modules rather than as distinct components.^9,24,25,26^ Furthermore, we do not have comparative data available prior to the 1-year safety program. We did not want to discourage resource-limited LTC facilities from joining the program due to the need for onerous retrospective data collection. Additionally, we cannot discount the possibility of inaccuracies with antibiotic data collection by participating sites. Our lack of access to protected health information precluded our ability to ensure the integrity of antibiotic use data submitted from hospitals or an evaluation of appropriate antibiotic use. Nonetheless, rigorous steps were followed to maximize the likelihood of valid data submission, as previously described.

Seasonal trends can play a role in antibiotic use, and while data are limited, there are reports of increased antibiotic use in LTC facilities during the winter months, with antibiotic use particularly driven by an increase in respiratory infections.^27^ Although seasonal trends may have had some impact on the high prescribing rates observed at the beginning of the safety program (in January), we compared these rates with data collected at the end of program (in December) where similar increases in antibiotic use in LTC are generally observed.^27,28^ No such increases were observed in our cohort.

Finally, changes in antibiotic usage identified in the safety program may have been affected by secular trends. The CMS mandated that LTC facilities have ASPs by 2017.^3,4,29,30^ However, if this mandate impacted our results, an uptake in ASPs would have likely affected both baseline data as well as data at the conclusion of the study.

## Conclusions

Developing, implementing, and sustaining ASPs in LTC settings can be challenging due to frequent staff turnover and limited time and resources. Despite these challenges, the AHRQ Safety Program used patient-safety principles, multidisciplinary education, and a multitude of interactive tools aimed at incorporating stewardship principles into daily practice; high participation in the program was associated with a reduction in antibiotic use and improved outcomes. Data are needed to evaluate the sustainability of these interventions and their long-term effect on antibiotic use, resident outcomes, and staff and resident satisfaction.

## Notes

Supplement. **eMethods 1.** Monthly Data Collection Form **eMethods 2.** Example of a Quarterly Benchmarking Report Provided to a Unit by the AHRQ Safety Program **eTable 1.** Detailed Summary of the Content of the AHRQ Patient Safety Program **eTable 2.** Total Antibiotic Starts per 1,000 Resident Days of Care (RD) **eTable 3.** Total Antibiotic Days of Therapy per 1,000 Resident Days of Care (RD) **eTable 4.** Antibiotic Starts per 1,000 Resident Days of Care (RD) for Selected Antibiotic Classes **eTable 5.** Antibiotic Days of Therapy per 1,000 Resident Days of Care (RD) for Selected Antibiotic Classes **eTable 6.** Urine Cultures per 1,000 Resident Days of Care **eTable 7.** *Clostridioides difficile* LabID Events per 10,000 Resident Days of Care **eFigure 1.** Reasons Long-term Care Sites Withdrew from the Safety Program **eFigure 2.** Distribution of 439 Long-term Care Settings Across the United States Enrolled in the AHRQ Safety Program **eFigure 3.** Top 20 Most Downloaded AHRQ Safety Program for Improving Antibiotic Use Materials During the Long-term Care Cohort **eFigure 4.** Participant Ratings of Usefulness of Webinars **eFigure 5.*** Clostridioides difficile* LabID Events per 10,000 Resident Days
