# Supplementary material for: Implementation of an Antibiotic Stewardship Program in Long-term Care Facilities Across the US
Source: JAMA Netw Open. 2022 Feb 28;5(2):e220181. doi: 10.1001/jamanetworkopen.2022.0181 (PMC8886516; doi:10.1001/jamanetworkopen.2022.0181)
Supplement: Supplement. — eMethods 1. Monthly Data Collection Form eMethods 2. Example of a Quarterly Benchmarking Report Provided to a Unit by the AHRQ Safety Program eTable 1. Detailed Summary of the Content of the AHRQ Patient Safety Program eTable 2. Total Antibiotic Starts per 1,000 Resident Days of Care (RD) eTable 3. Total Antibiotic Days of Therapy per 1,000 Resident Days of Care (RD) eTable 4. Antibiotic Starts per 1,000 Resident Days of Care (RD) for Selected Antibiotic Classes eTable 5. Antibiotic Days of Therapy per 1,000 Resident Days of Care (RD) for Selected Antibiotic Classes eTable 6. Urine Cultures per 1,000 Resident Days of Care eTable 7. Clostridioides difficile LabID Events per 10,000 Resident Days of Care eFigure 1. Reasons Long-term Care Sites Withdrew from the Safety Program eFigure 2. Distribution of 439 Long-term Care Settings Across the United States Enrolled in the AHRQ Safety Program eFigure 3. Top 20 Most Downloaded AHRQ Safety Program for Improving Antibiotic Use Materials During the Long-term Care Cohort eFigure 4. Participant Ratings of Usefulness of Webinars eFigure 5. Clostridioides difficile LabID Events per 10,000 Resident Days [file jamanetwopen-e220181-s001.pdf]

Supplemental Online Content

Katz MJ, Tamma PD, Cosgrove SE, et al. Implementation of an antibiotic stewardship program in long-term care facilities across the US. *JAMA Netw Open.* 2022;5(2):e220181. doi:10.1001/jamanetworkopen.2022.0181

eMethods 1. Monthly Data Collection Form

eMethods 2. Example of a Quarterly Benchmarking Report Provided to a Unit by the AHRQ Safety Program

eTable 1. Detailed Summary of the Content of the AHRQ Patient Safety Program

eTable 2. Total Antibiotic Starts per 1,000 Resident Days of Care (RD)

eTable 3. Total Antibiotic Days of Therapy per 1,000 Resident Days of Care (RD)

eTable 4. Antibiotic Starts per 1,000 Resident Days of Care (RD) for Selected Antibiotic Classes

eTable 5. Antibiotic Days of Therapy per 1,000 Resident Days of Care (RD) for Selected Antibiotic Classes

eTable 6. Urine Cultures per 1,000 Resident Days of Care

eTable 7. *Clostridioides difficile* LabID Events per 10,000 Resident Days of Care

eFigure 1. Reasons Long-Term Care Sites Withdrew from the Safety Program

eFigure 2. Distribution of 439 Long-term Care Settings Across the United States Enrolled in the AHRQ Safety Program

eFigure 3. Top 20 most downloaded AHRQ Safety Program for Improving Antibiotic Use Materials During the Long-term Care Cohort

eFigure 4. Participant Ratings of Usefulness of Webinars

eFigure 5. *Clostridioides difficile* LabID Events per 10,000 Resident Days

This supplemental material has been provided by the authors to give readers additional information about their work.

© 2022 Katz MJ et al. *JAMA Network Open.*

eMethods 1. Monthly Data Collection Form

Form Approved
OMB No. 0935-0238
Exp. Date 8/31/2022

General Instructions:

* Each LTC facility participating in the AHRQ Safety Program for Improving Antibiotic Use should use this spreadsheet to submit monthly data for:
    * monthly days of antibiotic therapy
    * monthly antibiotic starts
    * monthly *C. Difficile* Laboratory-identifiable (LabID) events
    * monthly urine culture data.
* Separate spreadsheets should be submitted for each reporting month
* Please speak with your Implementation Advisor regarding the timeline for the monthly data submissions
* Please **DO NOT** modify the structure of the spreadsheet such as adding, deleting, or altering rows or columns
* **Please fill in all non-colored cells**. All numbers are expected to be whole numbers.
* Please use the facility name that you used during website registration.

Instructions for Reporting:

Number of patient days of care: Sum of each daily patient census (number of residents in-house) for the reporting month. Other synonymous terms include "monthly resident-days", "monthly bed days of care", "monthly inpatient service days", "monthly occupied bed days", or "monthly census inpatient days of care".

***C. difficile* LabID Events**: Total number of non-duplicate *C. difficile* positive laboratory assay obtained while a resident is receiving care from your nursing home during the reporting month.
C. difficile positive laboratory assay: An unformed/loose stool that tests positive for *C. difficile* toxin A and/or B, (includes molecular assays [PCR] and/or toxin assays) OR a toxin-producing *C. difficile* organism detected in an unformed/loose stool sample by culture or other laboratory means.
Duplicate C. difficile positive laboratory assay: any *C. difficile* positive laboratory assay from the same resident following a previous *C. difficile* positive laboratory assay within the past two weeks [<15 days]. There should be at least 14 calendar days with no C. difficile positive laboratory assay for the resident before another *C. difficile* LabID Event is counted for the numerator. The date of specimen collection is considered Day 1. Please see Appendix 2 for *C. difficile* LabID events counting algorithm.

Urine Cultures Collected: Total number of urine culture obtained while a resident is receiving care from your nursing home during the reporting month.

Days of Antibiotic Therapy (DOT): Aggregate number of days residents were administered each of the antibiotics for the reporting month. For example, if your unit had 3 residents using Ciprofloxacin in January 2019 and they used it for 3, 5, & 7 days, respectively, then days of Ciprofloxacin in January 2019 should be counted as 3+5+7=15 days
* Please see NDC codes for corresponding antibiotics in Appendix 1.
* Please note that antibiotic brand names are listed only as examples. Not all brand names are listed.
* If you anticipate difficulties in collecting antibiotic usage data for the antibiotics listed below, please contact your Implementation Advisor for assistance.

Antibiotic Starts: Total number of antibiotic started among all housed residents for each antibiotic for the reporting month
* If a resident is transferred into your facility while on antibiotics, those antibiotics are also considered a 'start'.
* Please note that antibiotic brand names are listed only as examples. Not all brand names are listed.

© 2022 Katz MJ et al. *JAMA Network Open.*

Please upload completed form to the website portal using your log-in credentials or fax to: 410-500-4243; Attn: AHRQ Safety Program - LTC
If you are uploading completed form to the website portal, please be sure to save it with file name: [Facility name_Reporting month], e.g. ABC Nursing Home_Jan 2019 (please use the facility name that you used during website registration)

**NPI**	[enter NPI here]	
**Long-Term Care Facility Name**	[enter facility name here]	
**Contact Name**	[enter name here]	
**Contact Email**	[enter email here]	
**Contact Telephone Number**	[enter telephone number here]	
**Reporting Month**	[enter reporting month] To enter report month, use the arrow and dropdown list on the right.	
**Number of Patient days of care**	[the sum of each daily census (number residents in the facility each day) for the entire reporting month]	
**Number of *C. difficile* LabID events**	[enter # *C. difficile* LabID events for the reporting month]	
**Number of urine cultures collected**	[enter # urine cultures collected in the reporting month]	
	**Days of Antibiotic Therapy (DOT)** [enter aggregate # of days residents were administered each antibiotic below in the reporting month. If no resident was administered a particular antibiotic, enter "0" for that row]	**# Antibiotic Starts** [enter # of antibiotic starts for each antibiotic below in the reporting month. If there were no antibiotic starts for a particular antibiotic, enter "0" for that row]
AMIKACIN (Amikin)		
AMOXICILLIN (Amoxil), AMPICILLIN (Omnipen, Polycillin)		
AMOXICILLIN/CLAVULANATE (Augmentin)		
AMPICILLIN/SULBACTAM (Unasyn)		
AZITHROMYCIN (Zithromax)		
AZTREONAM (Azactam)		
CEFADROXIL (Duricef)		
CEFACLOR		
CEFAZOLIN (Ancef)		
CEFDINIR (Omnicef)		
CEFEPIME (Maxipime)		
CEFIXIME (Suprax)		
CEFPODOXIME (Vantin)		
CEFOTAXIME (Claforan)		
CEFTAROLINE (Teflaro)		
CEFTAZIDIME (Fortaz, Tazicef)		
CEFTRIAXONE (Rocephin)		
CEFUROXIME (Ceftin), CEFPROZIL (Cefzil)		
CEPHALEXIN (Keflex)		
CIPROFLOXACIN (Cipro)		
CLINDAMYCIN (Cleocin)		
DAPTOMYCIN (Cubicin)		
DOXYCYCLINE (Vibramycin)		
ERTAPENEM (Invanz)		
FOSFOMYCIN (Monurol)		
GENTAMICIN		
IMIPENEM (Primaxin)		
LEVOFLOXACIN (Levaquin), OFLOXACIN (Floxin)		
LINEZOLID (Zyvox), TEDIZOLID (Sivextro)		
MEROPENEM (Merrem)		
METRONIDAZOLE (Flagyl)		
MINOCYCLINE		
MOXIFLOXACIN (Avelox)		
NITROFURANTOIN (Macrobid)		
PIPERACILLIN/TAZOBACTAM (Zosyn)		
SULFAMETHOXAZOLE/TRIMETHOPRIM (Bactrim, Septra)		
TETRACYCLINE (oral forms only)		
TOBRAMYCIN (Tobrex)		
TRIMETHOPRIM		
VANCOMYCIN ***Intravenous*** (Vancocin)		
VANCOMYCIN ***Oral*** (Vancocin)		

Public reporting burden for this collection of information is estimated to average 60 minutes per response, the estimated time required to complete the survey. An agency may not conduct or sponsor, and a person is not required to respond to, a collection of information unless it displays a currently valid OMB control number. Send comments regarding this burden estimate or any other aspect of this collection of information, including suggestions for reducing this burden, to: AHRQ Reports Clearance Officer Attention: PRA, Paper Reduction Project (0935-0238) AHRQ, 5600 Fishers Lane, #07W41A, Rockville, MD 20857.

The confidentiality of your responses are protected by Sections 944(c) and 308(d) of the Public Health Service Act [42 U.S.C. 299c-3(c) and 42 U.S.C. 242m(d)]. Information that could identify you will not be disclosed unless you have consented to that disclosure

© 2022 Katz MJ et al. *JAMA Network Open.*

eMethods 2. Example of a Final Benchmarking Report Provided to a Unit by the AHRQ Safety Program

Quarterly Benchmarking Report, January to December 2019 (Q1-Q4)

1. Introduction

Facility: FACILITY NAME
Facility Type: Skilled Nursing Facility
Benchmark: all participating facilities with less than 100 certified beds

As part of participation in the AHRQ Safety Program for Improving Antibiotic Use, your facility receives quarterly benchmarking reports to compare your facility's progress to those of similar facilities.

This report contains individualized results from all the data submitted by your facility for the 1st, 2nd, 3rd, & 4th quarters (January-December 2019). It includes the following results for your facility, where data are available at the time of production of this report.

- 1st, 2nd, 3rd, & 4th quarters antibiotic days of therapy (DOT)
- 1st, 2nd, 3rd, & 4th quarters antibiotic starts
- 1st, 2nd, 3rd, & 4th quarters *C. difficile* LabID events
- 1st, 2nd, 3rd, & 4th quarters urine culture events
- Baseline and Endline Structural Assessment Data
- Baseline and Endline NHSOPS

This report also includes aggregate results from similar participating facilities (benchmarking facilities). This benchmark is the average of results from all benchmark facilities whose data are available at the time of production of this report. In addition to directly comparing your facility's results with the benchmark, we provide your facility's rank among benchmarking facilities. For instance, if your facility's rank is 5/111 for DOT in April, that means only 4 out of 111 benchmarking facilities had a lower level of DOT than your facility in April.

If your facility submitted data that are reported using an older version of the data collection template and/or if your facility's data are out-of-range (high rates in comparison to the benchmark), your data is excluded from the benchmark calculation as they are not directly comparable to the benchmark. Usable newly submitted Q4 data and resubmitted data by 2/15/2020 are incorporated in the Q4 report. Please see the individual results below for more detail.

Please note that results from individual facilities are not shared with other participating facilities; the report only includes aggregate benchmark data from other facilities. We welcome your feedback on the report. If you have any questions about the report or your facility's results, please contact your Implementation Adviser

© 2022 Katz MJ et al. *JAMA Network Open.*

2. Structural Assessment

Table 1 summarizes the data from the baseline and endline Structural Assessment forms completed by your facility at the time of program registration, and at the end of the program. It also includes comparative Structural Assessment data from all participating facilities with less than 100 certified beds.

Table 1. Structural Assessment Items from Your Facility and Benchmark, Baseline and Endline.

Item	Your Facility (Baseline)	Benchmark (Baseline)	Your Facility (Endline)	Benchmark (Endline)
Number of certified beds in facility	90	67	74	66
Proportion of residents in skilled beds	40%	41%	9%	52%
Proportion of residents in residential beds	-	-	78%	82%
Actively involved in QI projects in past 2 years	No	100% Yes	Yes	89% Yes
Number of days consultant pharmacist spends at facility each month	1	2	1	2.9
Facility has existing Antibiotic Stewardship Program (ASP)	Yes	100% Yes	No	96% Yes
AS activity: developing an antibiogram	No	0% Yes	No	44% Yes
AS activity: in-service training to nurses on antibiotic use	No	100% Yes	Yes	87% Yes
AS activity: developing antibiotic prescribing recommendations	No	0% Yes	No	54% Yes
AS activity: formulary restriction of some antibiotics	No	0% Yes	No	22% Yes
AS activity: post-prescription review of select antibiotics	No	0% Yes	No	61% Yes
AS activity: developing protocols for treatment of common infectious syndromes	No	100% Yes	No	74% Yes
Antibiotic starts used to measured antibiotic use	Yes	100% Yes	Yes	87% Yes
Antibiotic days of therapy used to measured antibiotic use	No	0% Yes	No	75% Yes
Defined daily doses used to measured antibiotic use	No	0% Yes	No	23% Yes

© 2022 Katz MJ et al. *JAMA Network Open.*

4. Antibiotic Days of Therapy

Figure 2 shows the trend of monthly antibiotic days of therapy per 1,000 patient-days in your facility. It includes data for all antibiotics reported by your facility. The benchmark rates represent an average across all participating facilities with less than 100 certified beds. Rates below the benchmark line indicate your facility's antibiotic days of therapy are lower than the benchmark. Rates above the benchmark line indicate your facility's antibiotic days of therapy are higher than the benchmark.

Figure 2. Antibiotic Days of Therapy per 1,000 Patient-Days, Through Q4 2019.

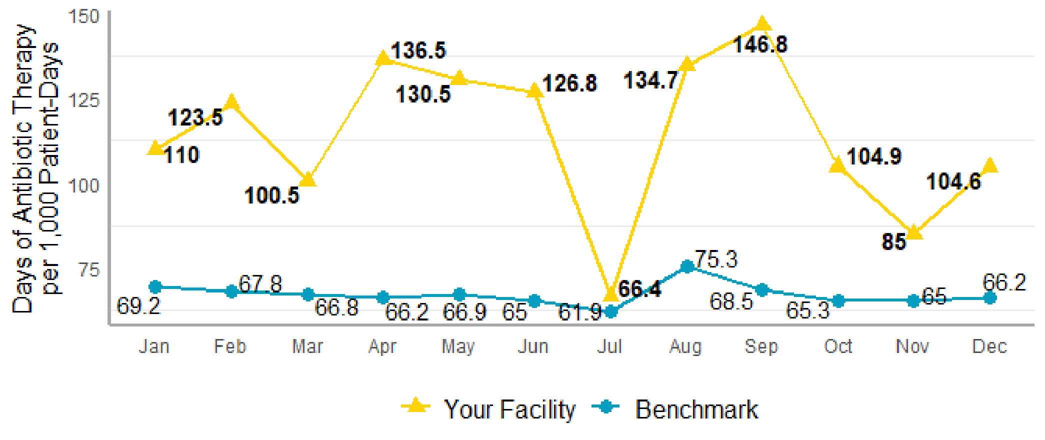

In addition, Table 2 shows how your facility ranks in this measure when compared with all facilities in the benchmark.

Table 2. Antibiotic Days of Therapy, Your Facility vs. Benchmark, Through Q4 2019.

Label	Jan	Feb	Mar	Apr	May	Jun	Jul	Aug	Sep	Oct	Nov	Dec
Total antibiotic days of therapy reported by your facility	233	234	204	278	273	259	134	254	261	196	148	187
Patient-days reported by your facility	2,119	1,895	2,030	2,037	2,092	2,042	2,017	1,885	1,778	1,868	1,741	1,787
Your facility's data are included in the benchmark	Yes	Yes	Yes	Yes	Yes	Yes	Yes	Yes	Yes	Yes	Yes	Yes
Your facility's rank among benchmarking facilities	149	168	149	163	159	163	120	155	151	136	126	133

© 2022 Katz MJ et al. *JAMA Network Open.*

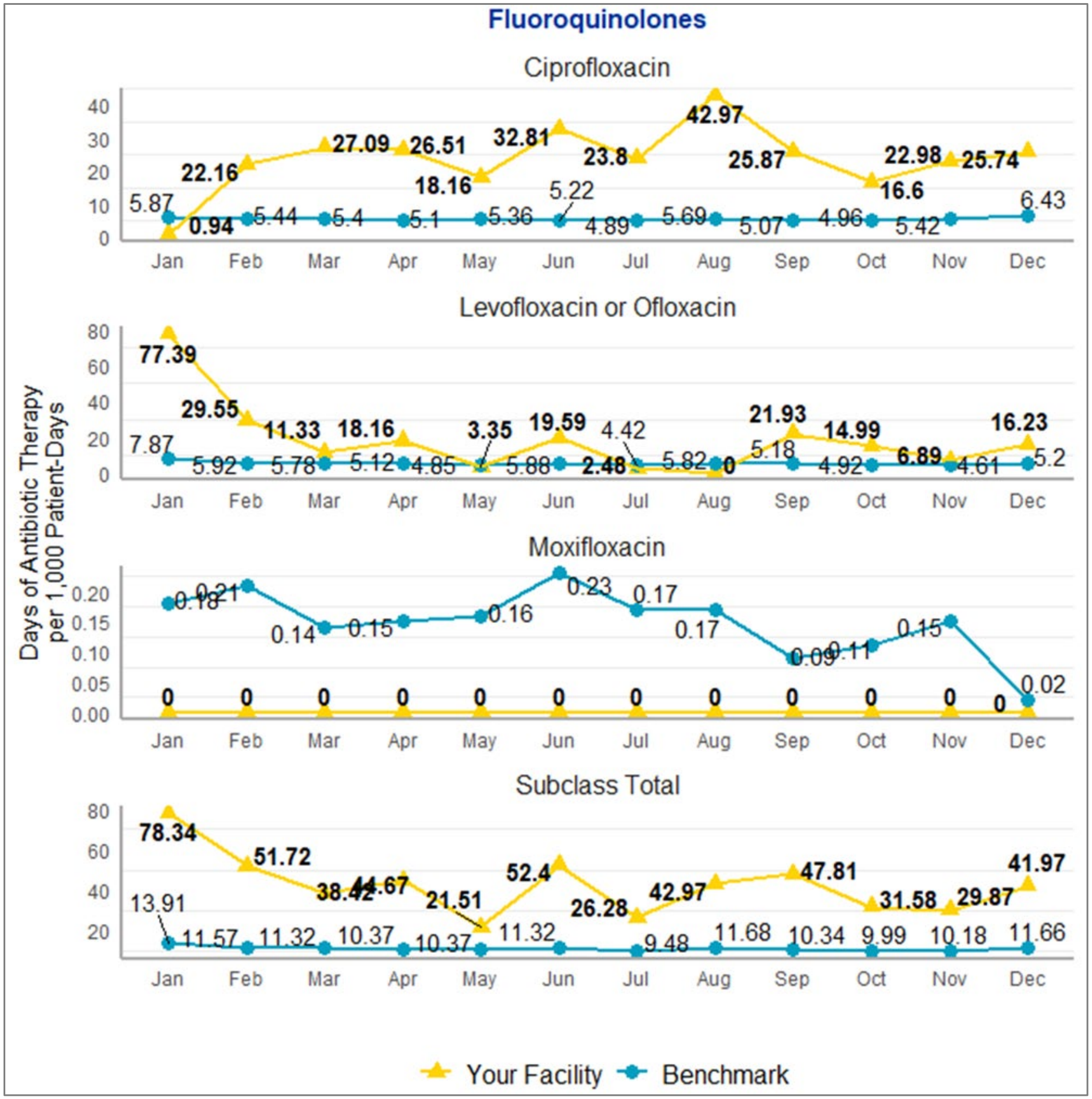

© 2022 Katz MJ et al. *JAMA Network Open.*

eTable 1. Detailed Summary of the Content of the AHRQ Patient Safety Program

Type of Tool/Specific Components	Goal	Name of Tool	Intended Audience	Frequency
**Webinars**				
Incorporating Antibiotic Stewardship Programs into Long-Term Care Settings	Introduce antibiotic stewardship principles and strategies to develop or enhance antibiotic stewardship programs in long-term care settings, including methods to measure and share outcomes.	Antibiotic stewardship program development specific to long-term care settings	Stewardship team	The first 2 weeks of the program[1,2,3]
Science of Antibiotic Safety	Provide information demonstrating that antibiotic use is a patient safety issue; improve teamwork and communication; and identify antibiotic-associated harm as well as develop solutions to prevent it.	Engaging with senior leadership to support implementation	Stewardship team, Frontline providers	Every two weeks during months 2-4 of the program[1,2,3]
		Making the case that improving antibiotic use is a patient safety issue		
		Improving communication and teamwork around antibiotic decision making		
		Identifying targets for improvement in antibiotic decision making		
		Making effective changes at the system level to improve antibiotic decision making		
Infectious Disease Syndromes	Provide guidance for best practices in the diagnosis and management of common infectious diseases syndromes using the Four Moments of Antibiotic Decision Making framework.	Collection of microbiologic specimens	Stewardship team, Frontline providers	Monthly during months 5-9 of the program[1,2,3]
		Assessment and treatment of residents with urinary tract infections		
		Assessment and treatment of residents with respiratory tract infections		
Communication	Provide guidance for incorporating antibiotic stewardship principles into communications with prescribers, residents, and their family members.	Communication among health care team members that guides antibiotic decision making and prevents resident harm	Stewardship team, Frontline providers	Monthly during months 10 and 11 of the program[1,2,3]

© 2022 Katz MJ et al. *JAMA Network Open.*

			Communication and education of residents and their family about potential risks associated with antibiotics		
	Sustainability	Support maintaining the stewardship work that occurred during the program that will continue after the one-year program is completed.	Sustaining stewardship activities in long-term care settings	Stewardship team	The last month of the program[1,2,3]
**Narrated Presentations**					
	Novel Topics	Provide guidance on targeted topics that were not addressed in the webinars.	Assessment and management of residents with suspected skin or soft tissue infections	Stewardship team, Frontline providers	Available on the project website for independent access.
			Approach to the patient with a penicillin allergy		
	Topics Addressed in Webinars	Reinforce and enrich the guidance on topics addressed by the webinars.	Appropriate collection of microbiological specimens	Stewardship team, Frontline providers	Available on the project website for independent access.
			Assessment and treatment of residents with suspected urinary tract infections		
			Assessment and treatment of residents with suspected respiratory tract infections		
			Discussing infectious concerns about residents with antibiotic prescribers		
			Discussing infectious concerns about residents with family members and caregivers		
**Program Implementation and Data Collection**					
		To support successful program implementation, maintenance, and data collection	Monthly data collection form	Stewardship team	Monthly
		The Antibiotic Stewardship Team is encouraged to use Four Moments of Antibiotic Decision-Making Form to review 5-10 residents each month. The residents reviewed should be individuals who are being evaluated for an infection.	4 Moments of antibiotic decision-making review form	Stewardship team, Frontline providers	Monthly

© 2022 Katz MJ et al. *JAMA Network Open.*

	Integrating the Four Moments into daily practice for frontline staff and prescribing clinicians should lead to improved antibiotic use and enhanced patient safety	Completion guide for the 4 moments of antibiotic decision making review form	Stewardship team	N/A
	To support successful program implementation, maintenance, and data collection	Implementation Guide	Stewardship team	N/A
		Sustaining your Antibiotic Stewardship Program Guide	Stewardship team	N/A
Group Activity				
	Creating a safety culture that values appropriate antibiotic prescribing and takes action when harm, or the potential for harm (near miss) related to antibiotics is detected	Gap Analysis	Stewardship team	N/A
		Checkpoint Tool	Stewardship team	N/A
		Staff Safety Assessment Form	Stewardship team, Frontline providers	N/A
		Learning from Antibiotic Associated Adverse Events	Stewardship team, Frontline providers	N/A
		Intervention Worksheet	Stewardship team	N/A
		Commitment Poster	Stewardship team, Frontline providers, Residents and visitors	Annually
Posters/Pocket Cards				
	Integrating the Four Moments into daily practice should lead to improved antibiotic use and enhanced patient safety	4 Moments of Antibiotic Decision Making posters/pocket cards	Frontline providers	N/A
		Four Questions to Ask Your Doctor or Nurse About Antibiotics	Residents and visitors	
	Effective communication with residents and family members requesting antibiotics may help them	Talking with Residents and Family Members About Antibiotics	Frontline providers	N/A

© 2022 Katz MJ et al. *JAMA Network Open.*

	understand the potential risks associated with antibiotic use.	Talking With Residents and Family Members About Urinary Tract Infections		
		Talking With Residents and Family Members About Respiratory Tract Infections		
		DESC Technique Poster		
	Reinforce key messages addressed in the webinars.	Collection of Microbiological Cultures Poster	Frontline providers	n/a
		Appropriate Collection of Urine Specimens Poster	Frontline providers	
		Minimum Criteria for Initiation of Antibiotics for Suspected UTI (8 x 11, one-sided)	Frontline providers	
		Pneumonia Pocket Card (8 x 10; one-sided)	Frontline providers	
		Management of Skin/Soft Tissue Infections (4 x 6; two-sided)	Frontline providers	
		Identifying Delirium (4 x 6; two-sided)	Frontline providers, Residents and visitors	

[1]All webinars were recorded and had an associated slide set and detailed script; could be accessed on the project website by all participants in the Safety Program at any time.
[2]Each webinar topic presented three times to account for different time zones.
[3]All webinars led by Morgan Katz, Theresa Rowe or Robin Jump to enhance continuity across the span of the Safety Program and to build ongoing relationships with participants.

© 2022 Katz MJ et al. *JAMA Network Open.*

eTable 2. Total Antibiotic Starts per 1,000 resident days of care (RD)

Facility subgroup		Jan-Feb	Mar-Apr	May-Jun	July-Aug	Sept-Oct	Nov-Dec	p-value
**Entire cohort**	Overall	7.89	7.67	7.42	7.38	7.53	7.48	0.020
**Facility size**	0-74 certified beds	7.88	7.40	7.40	7.53	7.44	7.42	0.221
	75-149 certified beds	8.46	8.27	7.74	7.59	7.82	7.83	0.011
	150 or more certified beds	6.72	6.73	6.79	6.76	7.04	6.8	0.805
**Affiliation**	Hospital-based	8.55	7.52	7.28	7.76	7.67	7.72	0.048
	Non-hospital based and owned by a larger system	8.33	8.20	8.03	7.85	7.98	8.23	0.684
	Non-hospital based and not owned by a larger system	6.78	6.77	6.4	6.34	6.66	6.08	0.010
**Short stay**	Less than 25%	7.07	7.09	6.88	6.83	7.06	6.91	0.492
	At least 25% and less than 50%	9.73	9.64	9.14	9.33	8.84	9.38	0.487
	At least 50% and less than 75%	9.08	8.32	7.49	8.26	8.41	8.59	0.378
	At least 75%	8.05	7.46	7.55	7.00	7.40	7.08	0.012
**Location**	Urban	6.83	6.87	6.46	6.59	6.72	6.61	0.432
	Suburban	8.88	8.37	8.29	8.04	8.42	8.40	0.143
	Rural	7.65	7.53	7.24	7.26	7.24	7.18	0.098

NOTE: p-value is for the difference between Jan-Feb and Nov-Dec

© 2022 Katz MJ et al. *JAMA Network Open.*

eTable 3. Total Antibiotic Days of Therapy per 1,000 resident days of care (RD)

Facility subgroup		Jan-Feb	Mar-Apr	May-Jun	July-Aug	Sept-Oct	Nov-Dec	p-value
**Entire cohort**	Overall	64.1	63.2	61.6	62.2	62.6	61.0	0.068
**Facility size**	0-74 certified beds	68.1	66.1	66.1	68.0	65.8	64.0	0.306
	75-149 certified beds	67.6	66.3	63	63.8	64.8	63.9	0.113
	150 or more certified beds	53.2	54	54	53.3	54.8	52.3	0.733
**Affiliation**	Hospital-based	67.9	62.9	61.3	65.3	61.6	61.5	0.168
	Non-hospital based and owned by a larger system	66.9	65.2	65.7	65.5	66.2	66.4	0.849
	Non-hospital based and not owned by a larger system	57.4	59.2	54.4	55.1	56.5	52.2	0.051
**Short stay**	Less than 25%	57.3	58.2	56.5	57.9	58.5	57.2	0.963
	At least 25% and less than 50%	78.9	83.2	79.3	81.5	76.9	76.5	0.596
	At least 50% and less than 75%	74.2	62.3	60.1	65.2	65.4	67.3	0.302
	At least 75%	66.6	62.4	62.7	58.6	61.2	56.8	0.005
**Location**	Urban	56.2	56.7	55.5	55.4	56.5	54.2	0.526
	Suburban	68.5	68.9	66.2	66.1	68.5	66.8	0.552
	Rural	65.1	62.1	61.1	62.9	61.1	60.2	0.075

NOTE: p-value is for the difference between Jan-Feb and Nov-Dec

© 2022 Katz MJ et al. *JAMA Network Open.*

eTable 4. Antibiotic Starts per 1,000 resident days of care (RD) for selected antibiotic classes

Antibiotic Class	Jan-Feb	Mar-Apr	May-June	July-Aug	Sept-Oct	Nov-Dec	p-value
Fluoroquinolones	1.495	1.426	1.355	1.296	1.326	1.280	0.002*
Penicillins (Non-Antipseudomonal)	0.898	0.887	0.871	0.829	0.894	0.860	0.393
Piperacillin / Tazobactam	0.089	0.081	0.108	0.094	0.113	0.107	0.128
Cephalosporins (1st Generation)	0.807	0.878	0.826	0.862	0.864	0.796	0.804
Cephalosporins (3rd Generation)	0.795	0.791	0.721	0.734	0.729	0.736	0.147
Cephalosporins (Antipseudomonal)	0.092	0.090	0.101	0.101	0.115	0.129	0.076
Glycopeptides	0.246	0.218	0.192	0.227	0.204	0.226	0.519
Macrolides	0.516	0.451	0.376	0.318	0.431	0.461	0.091
Sulfamethoxazole / Trimethoprim	0.636	0.603	0.594	0.602	0.614	0.628	0.836
Nitrofurantoin	0.388	0.381	0.426	0.375	0.367	0.366	0.429
Tetracyclines	0.696	0.643	0.667	0.673	0.674	0.703	0.901

NOTE: p-value is for the difference between Jan-Feb and Nov-Dec

© 2022 Katz MJ et al. *JAMA Network Open.*

eTable 5. Antibiotic Days of Therapy per 1,000 resident days of care (RD) for selected antibiotic classes

Antibiotic Class	Jan-Feb	Mar-Apr	May-June	July-Aug	Sept-Oct	Nov-Dec	p-value
Fluoroquinolones	10.60	9.84	9.57	9.50	9.41	9.41	0.014*
Penicillins (Non-Antipseudomonal)	7.29	7.28	7.34	6.67	6.93	6.97	0.415
Piperacillin / Tazobactam	2.18	2.31	2.58	2.32	2.80	3.01	0.103
Cephalosporins (1st Generation)	6.90	7.39	7.66	7.61	7.64	6.68	0.554
Cephalosporins (3rd Generation)	5.48	5.30	4.94	4.82	5.04	4.72	0.030*
Cephalosporins (Antipseudomonal)	1.41	1.43	1.54	1.74	1.81	2.19	0.031*
Glycopeptides	4.63	4.64	3.92	4.32	5.28	4.98	0.567
Macrolides	2.84	2.43	1.91	1.72	2.31	2.51	0.103
Sulfamethoxazole / Trimethoprim	5.54	5.64	5.26	5.73	5.66	5.52	0.950
Nitrofurantoin	4.48	4.54	4.41	4.10	4.06	3.95	0.160
Tetracyclines	7.10	6.70	7.17	7.40	7.16	7.45	0.447

NOTE: p-value is for the difference between Jan-Feb and Nov-Dec

© 2022 Katz MJ et al. *JAMA Network Open.*

eTable 6. Urine Cultures per 1,000 resident days of care

Facility subgroup		Jan-Feb	Mar-Apr	May-Jun	July-Aug	Sept-Oct	Nov-Dec	p-value
**Entire cohort**	Overall	3.01	2.73	2.74	2.79	2.73	2.63	0.001*
**Facility size**	0-74 certified beds	3.31	2.82	3.08	2.96	3.00	2.80	0.030*
	75-149 certified beds	3.07	2.84	2.69	2.87	2.70	2.60	0.001*
	150 or more certified beds	2.55	2.51	2.44	2.52	2.47	2.55	0.986
**Affiliation**	Hospital-based	3.34	3.04	2.97	2.86	2.66	2.72	0.009*
	Non-hospital based and owned by a larger system	3.17	2.86	2.93	2.99	2.89	2.82	0.037
	Non-hospital based and not owned by a larger system	2.58	2.37	2.28	2.39	2.45	2.23	0.063
**Short stay**	Less than 25%	2.76	2.64	2.52	2.58	2.63	2.58	0.237
	At least 25% and less than 50%	3.46	3.32	3.19	3.32	3.05	3.21	0.311
	At least 50% and less than 75%	3.34	2.71	3.01	2.84	2.91	2.67	0.105
	At least 75%	3.06	2.63	2.79	2.89	2.68	2.44	<0.001*
**Location**	Urban	2.55	2.65	2.53	2.49	2.33	2.37	0.265
	Suburban	3.48	2.88	3.12	3.14	2.98	2.85	0.001*
	Rural	2.81	2.71	2.51	2.65	2.77	2.62	0.284

NOTE: p-value is for the difference between Jan-Feb and Nov-Dec

© 2022 Katz MJ et al. *JAMA Network Open.*

eTable 7. *Clostridioides difficile* LabID events per 10,000 resident days of care

Facility subgroup		Jan-Feb	Mar-Apr	May-Jun	July-Aug	Sept-Oct	Nov-Dec	p-value
**Entire cohort**	Overall	1.66	1.65	1.09	1.59	1.29	1.50	0.524
**Facility size**	0-74 certified beds	1.05	1.03	0.51	1.77	0.72	0.63	0.217
	75-149 certified beds	1.91	2.19	1.41	1.61	1.5	1.68	0.583
	150 or more certified beds	1.89	1.39	1.18	1.52	1.52	2.25	0.467
**Affiliation**	Hospital-based	0.56	0.68	0.54	1.34	1.04	0.84	0.392
	Non-hospital based and owned by a larger system	2.05	2.03	1.36	1.99	1.63	2.11	0.884
	Non-hospital based and not owned by a larger system	1.48	1.43	0.86	0.94	0.78	0.87	0.109
**Short stay**	Less than 25%	1.14	1.33	0.77	0.94	0.84	1.01	0.618
	At least 25% and less than 50%	3.77	2.59	2.13	3.93	2.38	3.14	0.518
	At least 50% and less than 75%	1.38	1.78	1.57	2.05	1.66	2.02	0.469
	At least 75%	1.56	1.44	0.91	1.71	1.49	1.35	0.678
**Location**	Urban	2.17	1.83	1.16	1.08	0.85	1.01	0.009*
	Suburban	2.11	1.87	1.34	2.14	2.29	2.50	0.483
	Rural	1.00	1.32	0.84	1.45	0.81	1.08	0.807

NOTE: p-value is for the difference between Jan-Feb and Nov-Dec

© 2022 Katz MJ et al. *JAMA Network Open.*

eFigure 1. Reasons Long-Term Sites Withdrew From the Safety Program

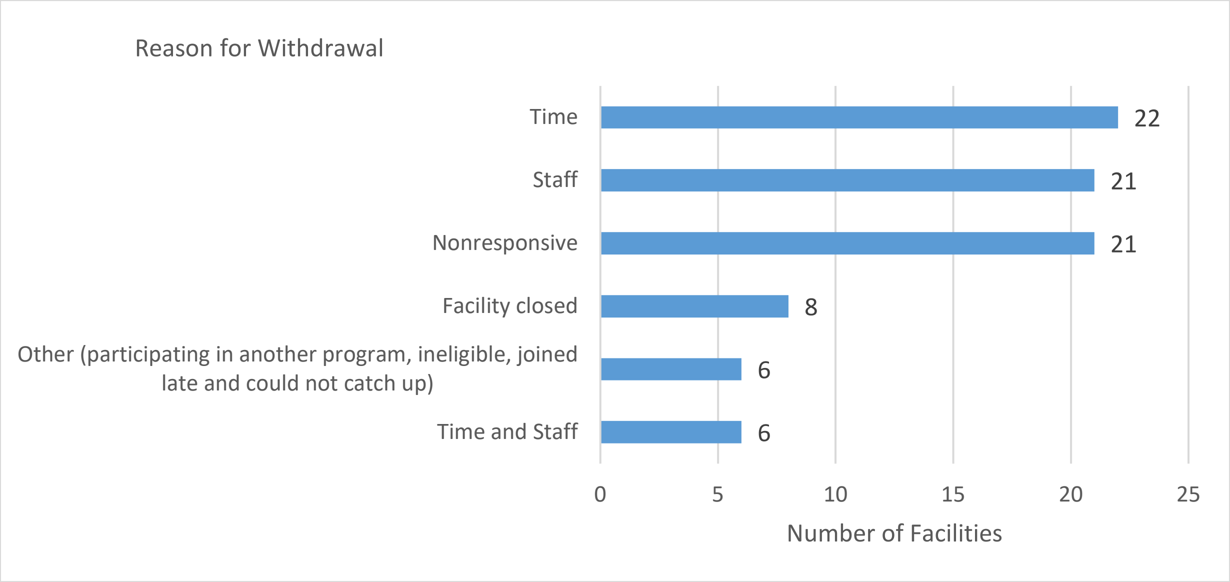

© 2022 Katz MJ et al. *JAMA Network Open.*

eFigure 2. Distribution of 439 long-term care settings across the United States enrolled in the AHRQ Safety Program

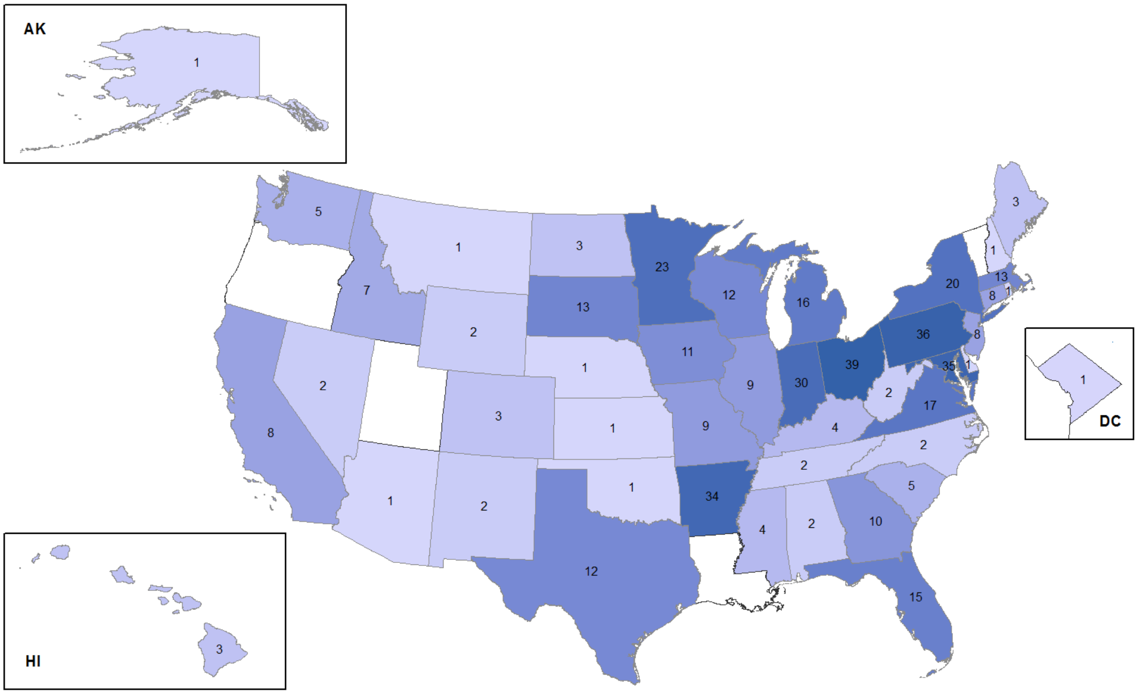

HHS Region	States & Territories	Total Number of Participating Facilities (after withdrawals)
1	Connecticut, Maine, Massachusetts, New Hampshire, Rhode Island, and Vermont	26
2	New Jersey, New York, Puerto Rico, and the Virgin Islands	28
3	Delaware, District of Columbia, Maryland, Pennsylvania, Virginia, and West Virginia	92
4	Alabama, Florida, Georgia, Kentucky, Mississippi, North Carolina, South Carolina, and Tennessee	45
5	Illinois, Indiana, Michigan, Minnesota, Ohio, and Wisconsin	129
6	Arkansas, Louisiana, New Mexico, Oklahoma, and Texas	48
7	Iowa, Kansas, Missouri, and Nebraska	22
8	Colorado, Montana, North Dakota, South Dakota, Utah, and Wyoming	23
9	Arizona, California, Hawaii, Nevada, USAPI (American Samoa, Commonwealth of the Northern Mariana Islands, Federated States of Micronesia, Guam, Marshall Islands, and Republic of Palau)	14
10	Alaska, Idaho, Oregon, and Washington	12
	TOTAL	439

© 2022 Katz MJ et al. *JAMA Network Open.*

eFigure 3. Top 20 most downloaded AHRQ Safety program for improving antibiotic use materials during the Long-Term Care Cohort.

**By the end of the Long-Term Care cohort, the 20 most popular materials on the Web site had just over 4,000 unique downloads (averaging 200 downloads per material).*

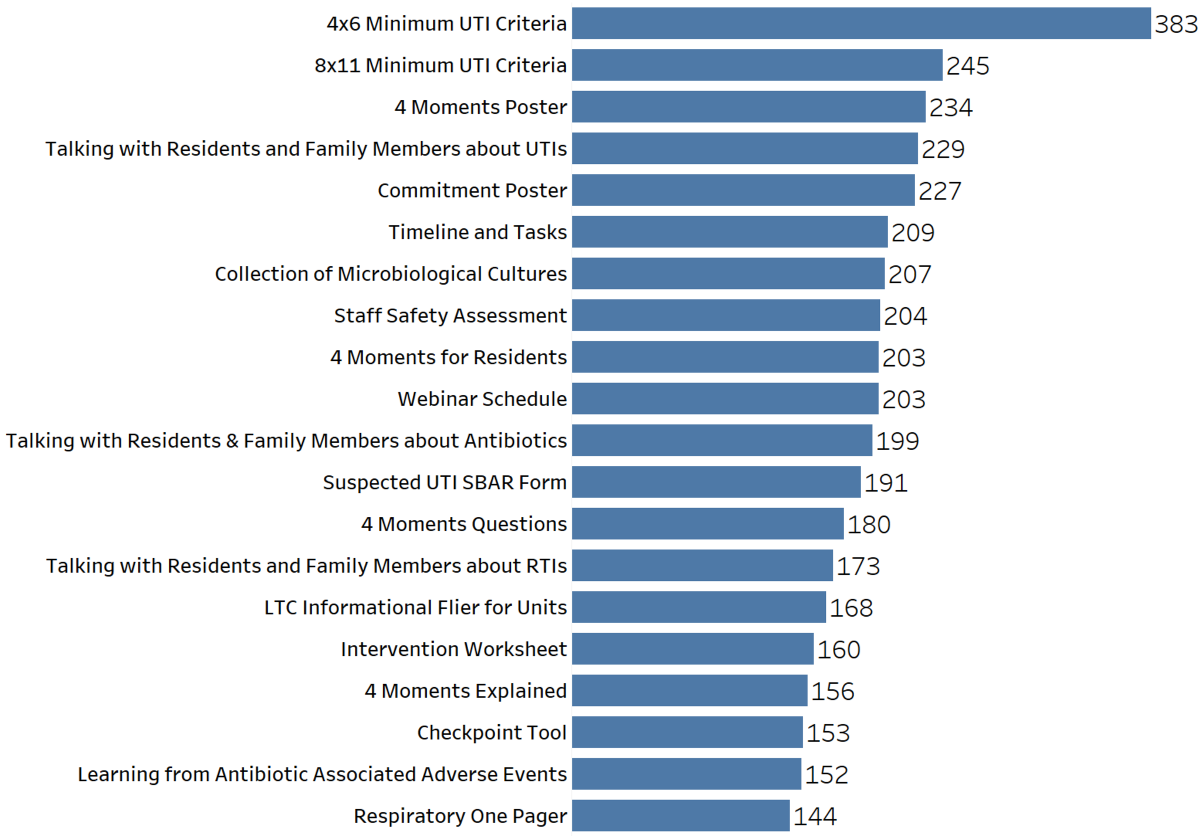

UTI = urinary tract infection; SBAR = Situation, Background, Assessment, and Recommendation; RTIs = respiratory tract infections

© 2022 Katz MJ et al. *JAMA Network Open.*

eFigure 4. Participant Ratings of Usefulness of Webinars

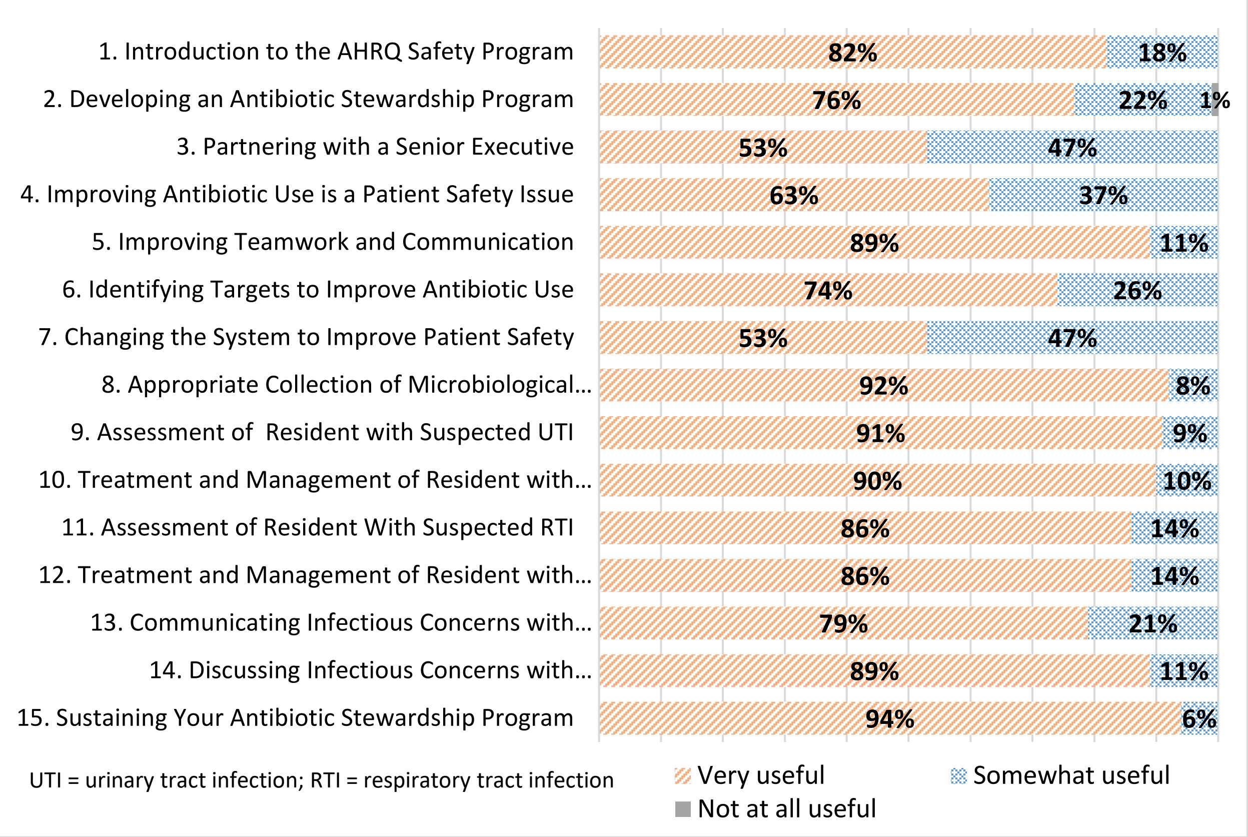

© 2022 Katz MJ et al. *JAMA Network Open.*

eFigure 5. ***Clostridioides difficile*** **LabID events per 10,000 resident days**

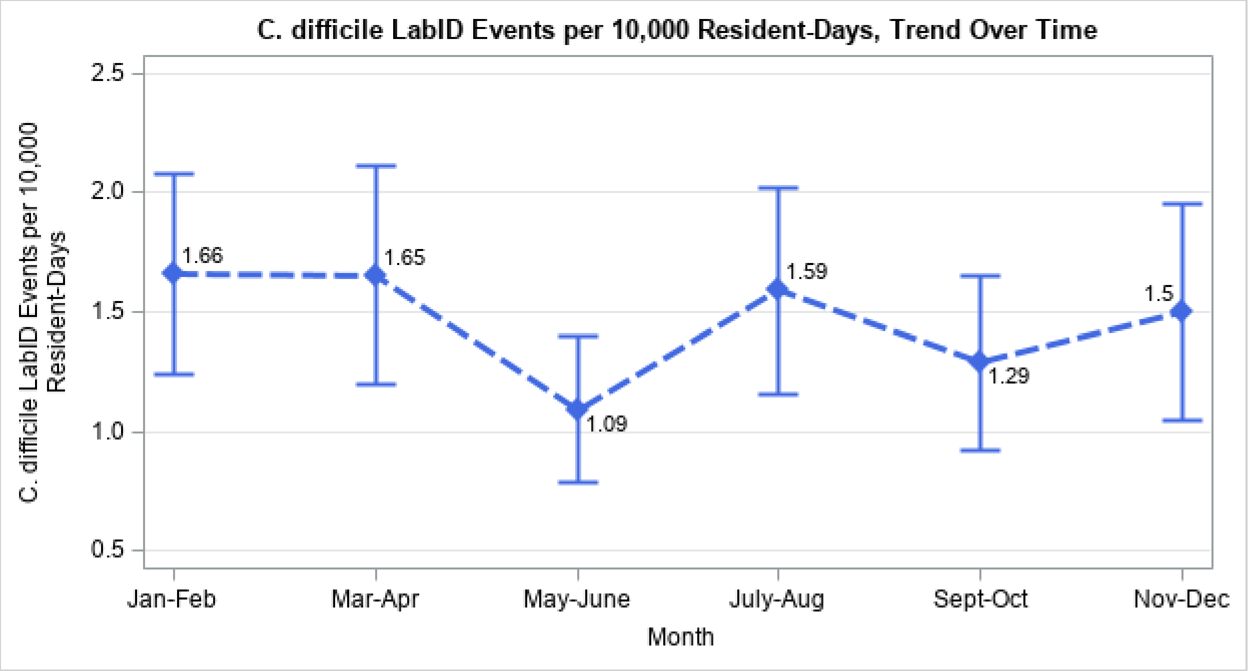

Graphs with Y axis starting at 0

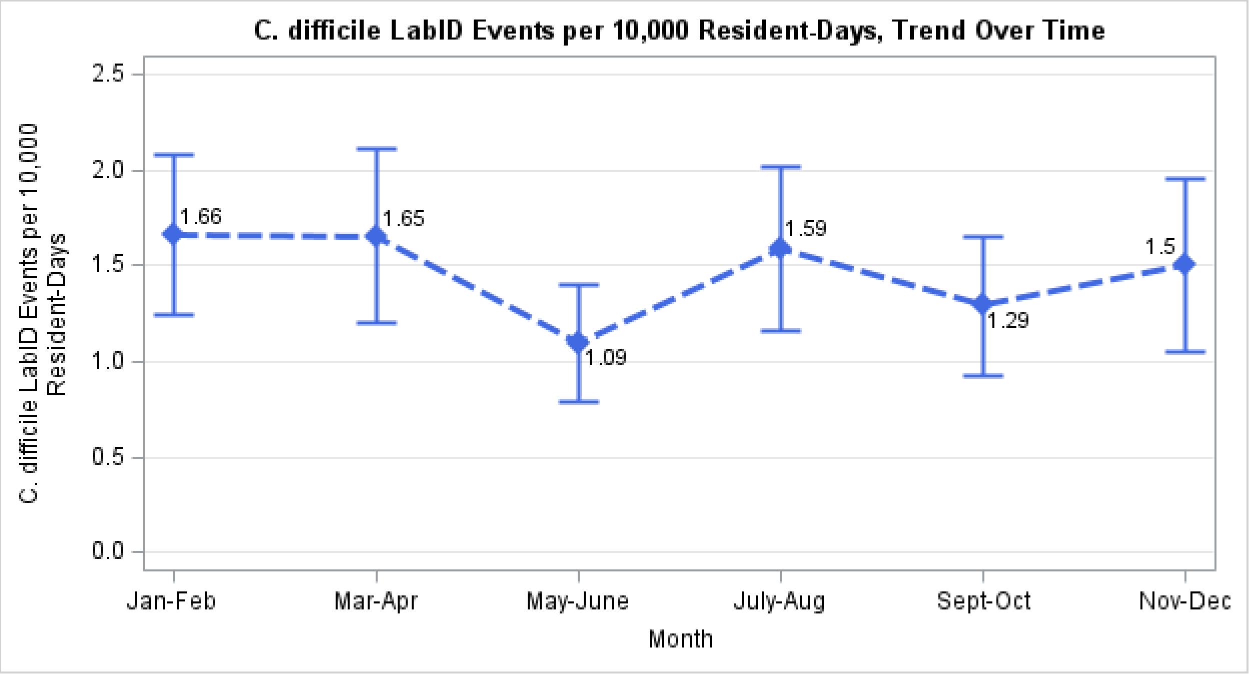

© 2022 Katz MJ et al. *JAMA Network Open.*
